# Ultrasound‐mediated mechanical forces activate selective tumor cell apoptosis

**DOI:** 10.1002/btm2.10737

**Published:** 2024-12-30

**Authors:** Ajay Tijore, Felix Margadant, Nehal Dwivedi, Leslie Morgan, Mingxi Yao, Anushya Hariharan, Claire Alexandra Zhen Chew, Simon Powell, Glenn Kunnath Bonney, Michael Sheetz

**Affiliations:** ^1^ Mechanobiology Institute National University of Singapore Singapore; ^2^ Department of Bioengineering Indian Institute of Science Bangalore Karnataka India; ^3^ Biochemistry and Molecular Biology Department University of Texas Medical Branch Galveston Texas USA; ^4^ Division of Hepatobiliary & Pancreatic Surgery, Department of Surgery National University Hospital Singapore; ^5^ ihealthtech National University Singapore Singapore

**Keywords:** apoptosis, cancer treatment, mechanical forces, Piezo1, ultrasound

## Abstract

Recent studies show that tumor cells undergo apoptosis after mechanical stretching, which promotes normal cell growth. Since ultrasound can produce similar sub‐cellular mechanical stresses on the nanoscale, here we test the effect of ultrasound‐mediated mechanical forces on tumors and normal cell survival. Surprisingly, tumor cells undergo apoptosis through a calpain‐dependent mitochondrial pathway that relies upon calcium entry through the mechanosensitive Piezo1 channels. This is a general property of all tumor cell lines tested irrespective of tissue origin, but normal cells are unaffected. In vivo, ultrasound treatment promotes tumor cell killing in a mouse model with invasive CT26 cancer cell subcutaneous tumors and in the chick chorioallantoic membrane (CAM) model with relatively minor damage to chick embryos. Further, patient‐derived pancreatic tumor organoids are killed by ultrasound treatment. Because ultrasound‐mediated mechanical forces cause apoptosis of tumor cells from many different tissues in different microenvironments, it may offer a safe, non‐invasive approach to augment tumor treatments.

**Translational Impact Statement**

We report that ultrasound‐mediated mechanical forces cause selective killing of cancer cells without significant damage to normal cells. This protocol is a dramatic improvement over previous attempts to thermally ablate tumors with ultrasound heating since our ultrasound treatment causes no heating in animal models and cell culture experiments while promoting killing. Thus, our results suggest that it is practical to develop ultrasound‐based therapies that could aid cancer treatment.

## 1 INTRODUCTION

Although mechanical perturbations of tissues benefit the organism and inhibit many disease processes, relatively little is known about how mechanical stresses affect cellular processes such as cancer. For example, exercise or stretching of tumors inhibits tumor growth in the mouse model.^1^, ^2^ Further, mechanically active muscle tissue has a low risk of tumor formation. In fact, muscle‐associated tumors do not even make it to the list of the most commonly occurring 36 tumors worldwide.^3^ At the cellular level, physiologically relevant fluid shear forces caused apoptosis in circulating and adherent tumor cells.^4^, ^5^ Further, our earlier studies show inhibition of growth and apoptosis of many different adherent tumor cells after mechanical stretching at a physiological level.^6^ The stretch‐mediated apoptosis depends on the mechanosensitive Piezo1 channel activity that enables calcium entry upon mechanical activation, initiating a process of calpain‐dependent apoptosis. In a broader context, normal cells can become transformed like tumor cells upon the depletion of rigidity sensing activity through the depletion of protein components of the rigidity sensor.^7^, ^8^ Surprisingly, such transformed normal cells from several tissues become mechanosensitive and apoptotic after stretching.^6^ Since most, if not all, tumor cells are depleted of rigidity sensors; mechanosensitivity appears to be a general feature of the tumor cells irrespective of tissue origin.^9^, ^10^ A much more flexible way to mechanically perturb cells is a low‐frequency ultrasound that can penetrate tissue easily. Here, we show that it provides a flexible way to inhibit tumor cell growth even in an organism.

In the past, ultrasound was used to sensitize tumor cells in combination with different strategies such as sonodynamic therapy,^11^ chemotherapy,^12^ and hyperthermia.^13^ In addition, different ultrasound modes have been tested individually to treat tumors using high‐intensity focused ultrasound (HIFU),^14^ high‐intensity pulsed ultrasound,^15^ and low‐intensity pulsed ultrasound.^16^ However, concerns were raised about healthy tissue damage surrounding the target area; hence, these methods found limited clinical use. Since many tumor cells appear mechanically vulnerable, we tested the effects of relatively low‐level ultrasound‐mediated mechanical forces that do not heat or otherwise damage healthy cells.

In this study, we find significant tumor cell killing by ultrasound‐mediated mechanical forces under conditions that do not damage the normal cells but penetrate tissue and apply mechanical stresses at the nanoscale on subcellular organelles.^17^ Many ultrasound applications have been approved for human use, and there is little evidence of damage to normal tissues by ultrasound.^18^, ^19^, ^20^ Because ultrasound‐mediated forces kill tumor cells, tumors and tumor organoids from many different tissues in a frequency range and at power levels approved for human use, ultrasound provides a potentially feasible way to attack tumor growth.^19^, ^20^, ^21^

## 2 RESULTS

### 2.1 Ultrasound induces tumor cell apoptosis

**FIGURE 1:**
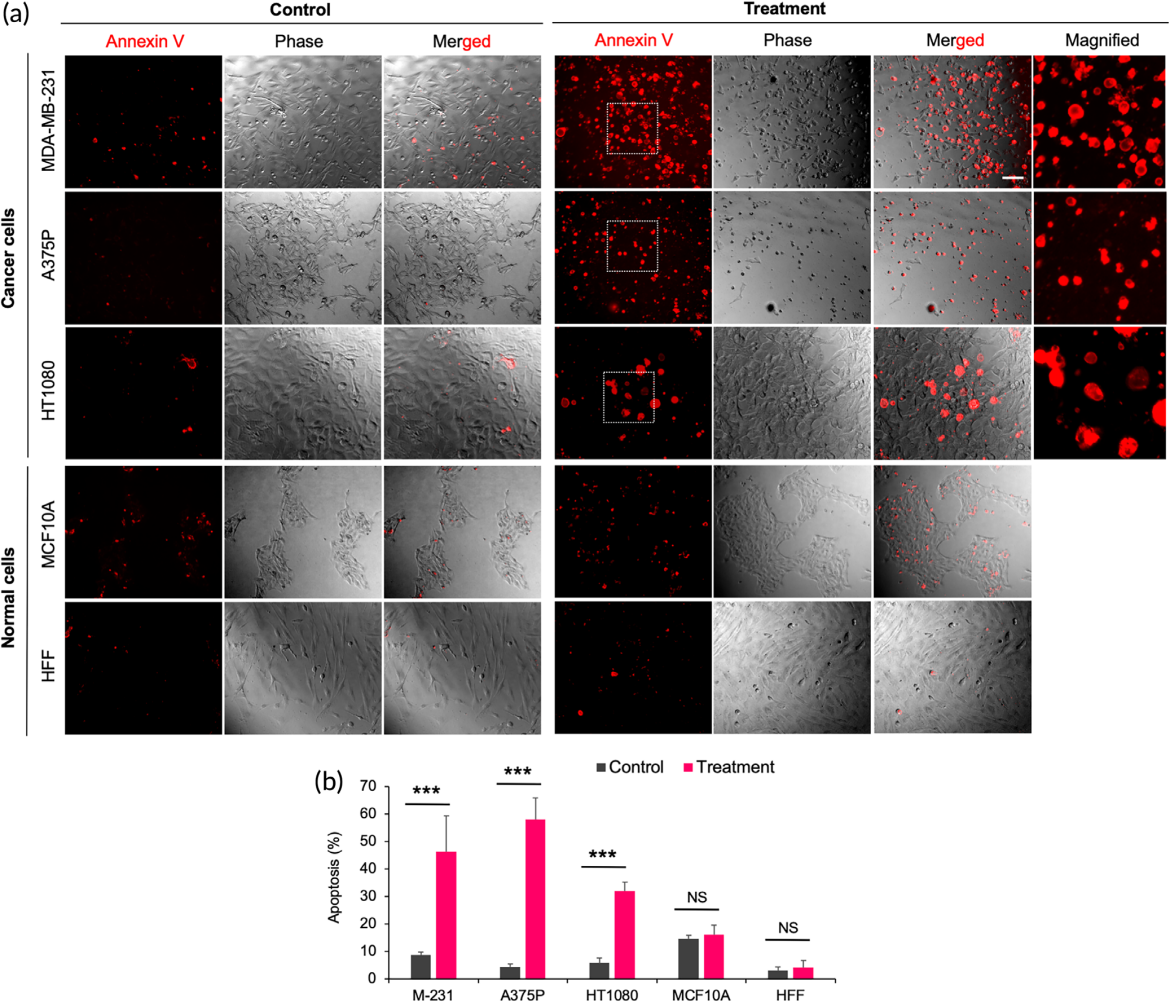
Ultrasound treatment (33 kHz) promotes apoptosis in tumor cells grown on matrigel. (a) Image panels showing apoptosis level in tumor cells (MDA‐MB‐231, A375p, and HT1080) and normal cells (MCF10A, HFF) with and without ultrasound treatment, scale bar: 50 μm. Magnified views of apoptotic cells were shown separately. (b) Bar diagram illustrating apoptosis level in tumor and normal cells with and without ultrasound treatment. *n* > 600 cells, data represent two independent experiments, ANOVA test, ***p < 0.001.

First, we tested the efficacy of ultrasound in inducing tumor cell apoptosis in vitro. Tumor cells were grown on matrigel and subjected to low‐frequency ultrasound (33 kHz) for 2 h, having a power level of 39 mW/cm^2^. A significant increase in apoptosis (46% for MDA‐MB‐231, 58% for A375p, and 32% HT1080) as measured by annexin V binding was observed with a 50% duty cycle in comparison to the non‐treated tumor cells (9% for MDA‐MB‐231, 4% for A375p, and 5% HT1080) (Figure 1a,b). In contrast, when normal cells from the same tissue of origin as the tumor cells were subjected to similar ultrasound treatment, negligible apoptosis was observed (16% for MCF10A and 3% for HFF after ultrasonication vs. 15% for MCF10A and 4% for HFF for non‐treated controls).

Since the ultrasound could also cause necrosis, we measured the level of necrosis. About 8%–9% of tumor cells (MDA‐MB‐231 and A375p) displayed both apoptosis plus necrosis upon treatment in comparison to 32%–35% of cells showing only apoptosis (Figure S1). To understand the role of ultrasound frequency in promoting apoptosis, experiments were repeated using another ultrasound frequency (120 kHz) with the same power level settings. However, there was no increase in tumor cell apoptosis upon treatment, unlike with 33 kHz frequency (Figure S2). To test the generality of apoptosis, tumor cells from another tissue (SKOV3 ovarian adenocarcinoma) were treated with ultrasound. An increase in apoptosis (32%) was seen in comparison to the non‐treated tumor cells (7%) (Figure S3). Thus, results showed that low‐frequency ultrasound‐mediated mechanical forces caused tumor cell apoptosis compared to relatively high‐frequency ultrasound in many tumor cell lines, which was reminiscent of the effects of cyclic mechanical stretch.^6^

Next, to measure the apoptosis levels and to test the differential killing of tumor cells at different power levels, we treated cells using a low‐power dose (1.5 W total output) and a high‐power dose (15 W total output) for 2 h (Figure S4a). A significant increase in tumor cell apoptosis was yet again observed at low power (13% for MDA‐MB‐231 and 12% for HT1080 after treatment vs. 7% for MDA‐MB‐231 and 3% HT1080 without treatment) (Figure S4b). As expected, matched tissue normal cells showed negligible apoptosis levels after similar treatment (14% for MCF10A and 2% for HFF after treatment vs. 10% for MCF10A and 2% for HFF without treatment). However, massive apoptosis was observed at high power in both cancer and normal cells (72% for MDA‐MB‐231, 63% for HT1080 vs. 80% MCF10A, and 61% HFF), highlighting the importance of power level while preserving differential killing cancer cells without damaging normal cells.

### 2.2 Repetitive ultrasound exposure results in a similar level of apoptosis each time

**FIGURE 2:**
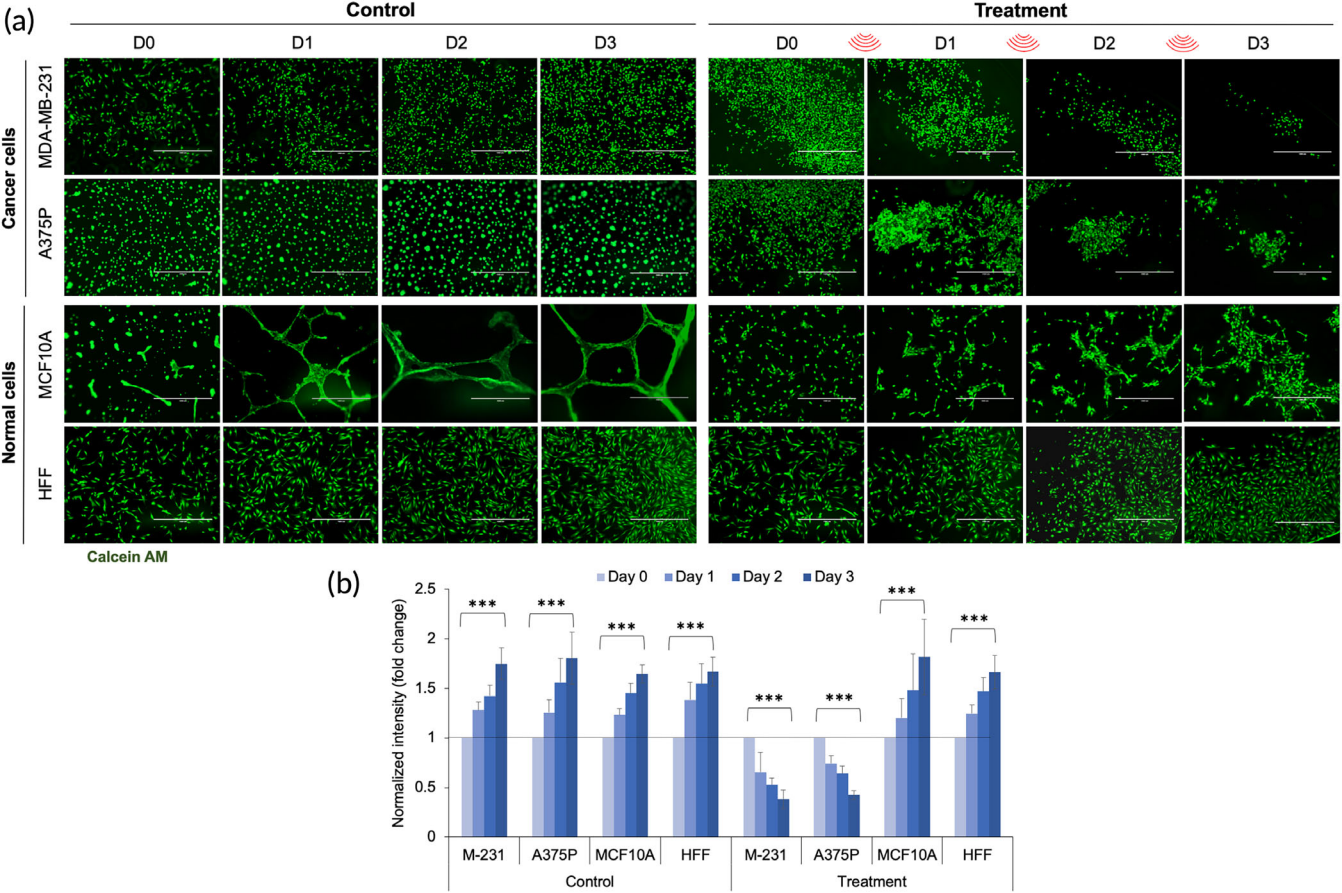
Repetitive ultrasound treatment (33 kHz) reduces tumor cell growth on matrigel. (a) Panels showing viable tumor and normal cells with and without repetitive ultrasound treatment from day 0 to day 3. Calcein AM was used to stain viable cells. Scale bar: 1000 μm. (b) Bar diagram showing normalized fluorescence intensity in tumor and normal cells in the control and ultrasound‐treated cells from day 0 to day 3. Day 0 intensity was considered one and shown by a dotted line. *n* > 5 image fields, data represent two independent experiments, ANOVA test, ***p < 0.001.

To test if an ultrasound‐resistant population of tumor cells could be produced by repeated ultrasound treatments, cells were treated with ultrasound (33 kHz) for 2 h/day for three consecutive days, having a power level of 39 mW/cm^2^. Viable cells were followed with calcein AM dye. A marked decrease in the tumor cell population (MDA‐MB‐231 or A375p) was observed after each round of treatment with no indication of a plateau. Further, the number of remaining viable cells decreased exponentially (Figure 2a). In contrast, non‐treated tumor cells showed progressive growth with time. Treatment of normal cells (MCF10A and HFF) caused no significant change in growth rate compared to non‐treated cells (Figure 2a). Further, fluorescence intensity measurements showed a significant decrease in the tumor cell population after treatment compared to the non‐treated tumor cells (Figure 2b). Thus, successive ultrasound treatment showed a similar level of tumor cell apoptosis each time, producing a dramatic overall decrease in tumor cell number.

### 2.3 Piezo1 channel mediated‐calcium influx is required for tumor cell apoptosis

**FIGURE 3:**
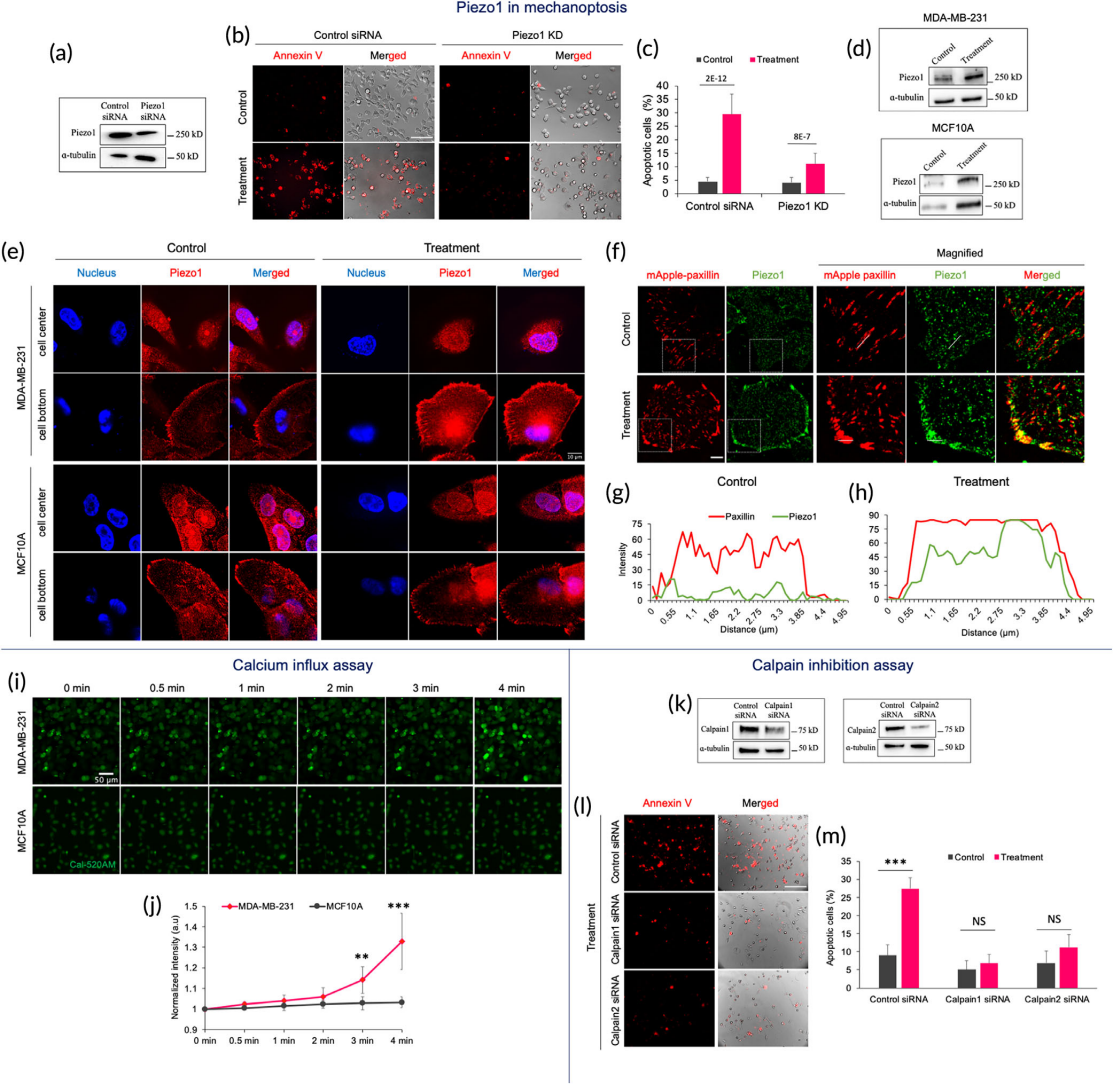
Ultrasound (33 kHz) enhances calcium influx through activated Piezo1 channels in tumor cells. (a) Western blot showing Piezo1 expression in control siRNA‐ and Piezo1 siRNA‐treated MDA‐MB‐231 cells. (b) Representative images displaying apoptotic cells with and without US treatment in control siRNA‐ and Piezo1 siRNA‐treated tumor cells. Scale bar: 100 μm. (c) Bar diagram exhibiting a level of apoptosis in tumor cells under different conditions, *n* > 1000 cells, Student's *t*‐test, ***p < 0.001. (d) Western blots show Piezo1 expression in MDA‐MB‐231 and MCF10A cells with and without US treatment. (e) Confocal images displaying spatial distribution of Piezo1 at the cell center and bottom in MDA‐MB‐231 and MCF10A cells with and without US treatment. Scale bar: 10 μm. (f) Representative images showing the spatial distribution of paxillin and Piezo1 in MDA‐MB‐231 cells with and without ultrasound treatment. Scale bar: 10 μm. (g,h) Fluorescent intensity plot displaying paxillin and Piezo1 distribution in cells with and without ultrasound treatment. (i) The time‐lapse montage displaying Cal‐520 AM dye intensity upon treatment in MDA‐MB‐231 and MCF10A cells. Scale bar 50 μm. (j) Graph representing Cal520 AM dye intensity plot of tumor and normal cells, *n* = 5 image fields from two experiments, Student's *t*‐test, **p < 0.01, ***p < 0.001. (k) Western blot showing calpain1 and 2 expressions after siRNA knockdown in tumor cells. (l) Representative images showing apoptotic tumor cells with ultrasound treatment in the presence of control siRNA, calpain1 siRNA and calpain2 siRNA. Scale bar: 100 μm. (m) Bar diagram displaying the extent of apoptosis in the presence of different siRNAs with and without ultrasound treatment. *n* > 1000 cells, data represent two experiments, Student's *t*‐test, ***p < 0.001.

The stretch‐induced tumor cell apoptosis (mechanoptosis) relied upon the mechanosensitive Piezo1 channels that catalyzed calcium entry,^22^ and we postulated that ultrasound‐induced apoptosis involved a similar mechanism.^6^ To test the role of Piezo1 in ultrasound‐mediated apoptosis, Piezo1 knock‐down (KD) cells were treated with ultrasound, and the apoptosis level was measured (Figure 3a,b). Interestingly, the apoptosis level was low in Piezo1 KD cells compared to control siRNA cells upon ultrasound treatment (33 kHz) for 2 h, having a power level of 39 mW/cm^2^ (Figure 3c). Thus, as found for stretch‐induced apoptosis, Piezo1 was also required for ultrasound‐based mechanoptosis.

Although there was a similar expression level of Piezo1 in different tumor cells and their matched normal counterparts^6^ (Figure S5), it was possible that the Piezo1 level increased with similar ultrasound treatment. To test this possibility, cells (MDA‐MB‐231 and MCF10A) were treated using ultrasound with identical parameters. Surprisingly, western blot results showed increased Piezo1 expression in tumor cells upon ultrasound treatment but not in normal cells (Figure 3d). Further, to determine if ultrasound caused changes in the spatial distribution of Piezo1, high‐resolution imaging was performed. In tumor cells, Piezo1 movement to the plasma membrane was observed following ultrasound treatment from a diffuse distribution in the cytoplasm (Figure 3e). In stark contrast, in normal cells, Piezo1 was predominately located in the nuclear region (especially the nuclear envelope)^6^ with and without ultrasound treatment. In particular, no redistribution of Piezo1 to the plasma membrane was detected in normal cells upon treatment, unlike tumor cells. These observations were further confirmed by wide‐field imaging. In particular, a 55% reduction in the nuclear to cytoplasmic intensity ratio was observed in treated tumor cells compared to non‐treated ones. In contrast, in normal cells, there was a 29% reduction in the ratio upon treatment (Figure S6). Recent studies have reported the association of Piezo1 with peripheral mature adhesion.^23^, ^24^ Hence, we examined if ultrasound treatment (33 kHz) promoted Piezo1 translocation to the peripheral adhesions in the tumor cells' plasma membrane. Cells transiently transfected with mApple‐paxillin were used to determine the adhesion distribution. Piezo1 was mostly diffuse in the cytoplasm in control tumor cells with no sign of association with peripheral adhesions (Figure 3f,g). In contrast, ultrasound‐treated tumor cells showed a strong association of Piezo1 with the enlarged adhesions at the cell periphery (Figure 3f,h). Thus, results demonstrated that ultrasound treatment promoted Piezo1 expression and its localization to the mature adhesions at the cell periphery in tumor cells. At the same time, it was primarily confined to the nuclear region in normal cells.

Based on the above results, we postulated that ultrasound‐mediated forces may trigger calcium influx through Piezo1 in tumor cell plasma membranes. In contrast, the nuclear distribution of Piezo1 in normal cells was consistent with the possibility that Piezo1 may not alter calcium entry through the plasma membrane upon ultrasound treatment. To test our hypothesis, an ultrasound‐induced calcium influx assay was performed. Cells were loaded with calcium‐sensitive dye Cal‐520AM to quantify the intracellular calcium level and subjected to ultrasound treatment (Figure 3i and Video S1). In tumor cells, a significant increase in the fluorescence intensity was observed 3 min after the treatment. In contrast, no such increase in the intensity was observed in normal MCF10A cells (Figure 3j and Video S2).

The role of calpain in mitochondria‐dependent apoptosis has been widely established. Hence, we tested the role of the calcium‐activated calpain protease in mechanoptosis. To do that, calpain 1 and 2 were knocked down in tumor cells by siRNAs (Figure 3k). Cells treated with scrambled siRNA were used as the control. After ultrasound treatment, calpain 1 and 2 knockdown cells had remarkably low levels of apoptosis compared to the ultrasound‐treated tumor cells (Figure 3l,m), indicating that calpain played a significant role in mechanoptosis. Collectively, the results demonstrated that ultrasound‐initiated calcium influx through activated Piezo1 channels acted as an upstream activator of mechanoptosis. Calcium activated‐calpain subsequently triggered mitochondria‐mediated apoptosis through a chain of reactions as described previously.^6^ In stark contrast, normal cells were unaffected by ultrasound‐mediated mechanical forces because ultrasound did not cause a dramatic rise in cytosolic calcium levels.

### 2.4 Ultrasound impedes in vivo tumor growth in the CAM tumor model

**FIGURE 4:**
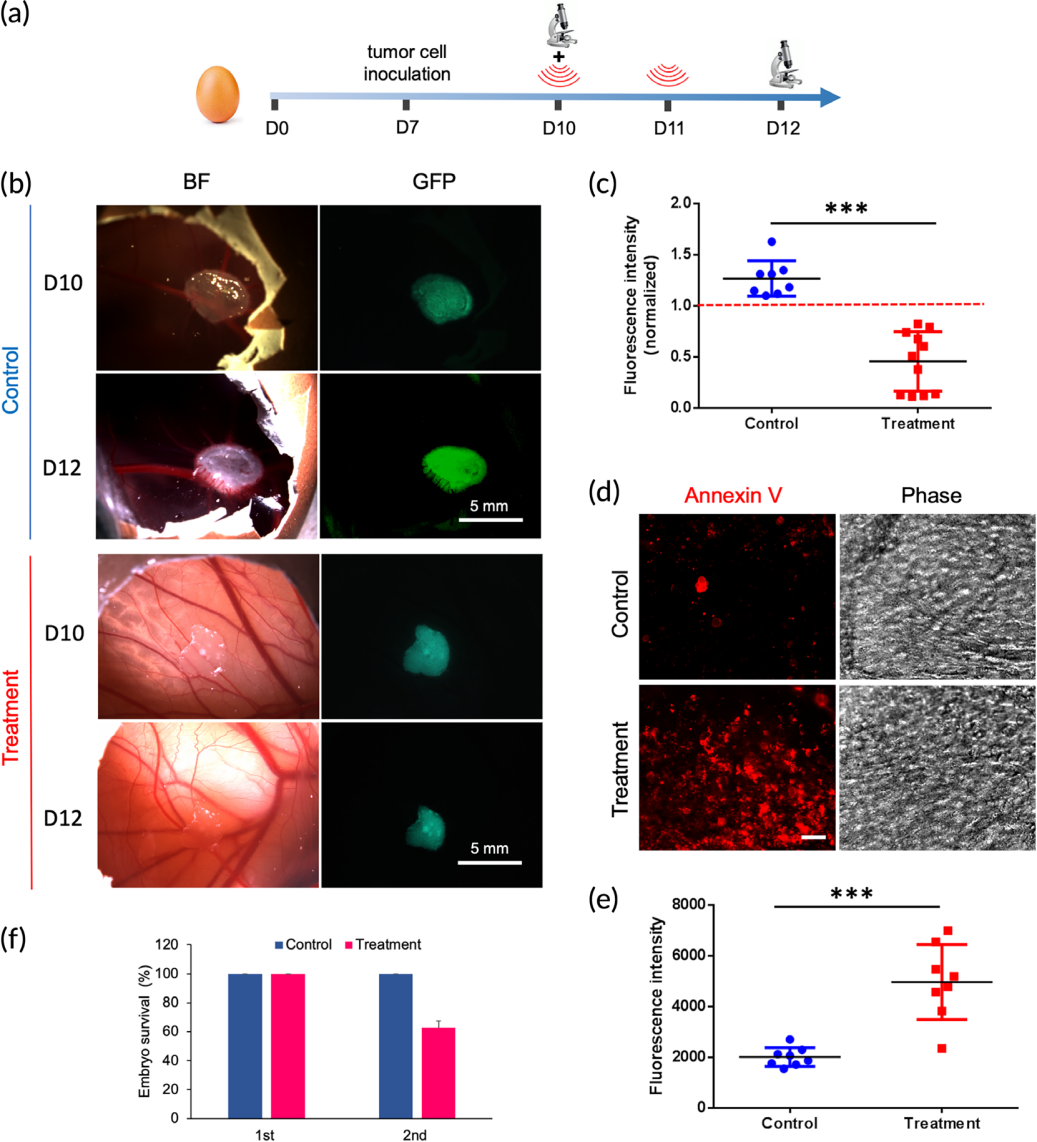
Ultrasound (33 kHz) causes a reduction in MDA‐MB‐231 tumor growth in the CAM model. (a) Time‐line depicting the scheme of ultrasonication of the tumors grown in the chick embryo model. (b) The top two panel rows display bright‐field and GFP expression images of the tumor before (D10) and after two rounds (D12) of ultrasound treatment. The bottom panel rows show bright‐field and GFP expression images of the tumor without any treatment on D10 and D12. (c) Graph showing fluorescence intensity of tumors with and without ultrasound treatment on D12. A red dotted line shows baseline intensity. (*n* = 8 for control and *n* = 11 for treatment) data represent three independent experiments, Student's *t*‐test, ***p < 0.001. (d) Image panels showing annexin‐V‐stained apoptotic cells in CAM tumors with and without treatment. (e) Graph showing annexin‐V intensity of CAM tumors with and without ultrasound treatment. (*n* = 8 image fields from control and treated samples), Student's *t*‐test, ***p < 0.001. (f) Bar diagram showing chick embryo survival rate after two rounds of ultrasound treatment (*n* = 13).

Since ultrasound killed tumor cells grown on the matrigel, we hypothesized whether ultrasound treatment would promote tumor killing in vivo without causing noticeable damage to healthy tissues. To test the hypothesis, we inoculated chick chorioallantoic membranes (CAM) with stable GFP‐transfected MDA‐MB‐231 tumor cells in a matrigel plug and allowed them to grow for 2 days. After tumors were established (often with vascularization), the embryos were treated with ultrasound (33 kHz) for 2 h/day for two consecutive days, having a power level of 39 mW/cm^2^ (Figures 4a and S7). Consistent with the in vitro results, we found an average two‐fold reduction in GFP fluorescence intensity after 2 days, indicating cell killing in the treated tumors. In contrast, non‐treated tumors displayed an increase in intensity, indicating normal tumor growth (Figure 4b,c). Next, we performed an annexin V‐based apoptosis assay on carefully excised ultrasound‐treated and controlled tumors. Consistent with in vitro results, treated tumors had a high fraction of apoptotic cells (Figure 4d,e). However, in the control tumors, few apoptotic cells were observed. In addition, the viability of chick embryos was assessed after every round of ultrasound treatment. There was a 100% survival rate after the first round of ultrasound (Figure 4f). After the second round of ultrasound, chick embryo survivability was reduced to 60%, while it remained at 100% in untreated embryos. Thus, these findings indicated that ultrasound treatment killed tumor cells in the chick embryo while causing less damage to delicate embryonic tissues/organs.

To determine if other transformed cells behaved similarly, we used HEK293T cells stably transformed by SV40 large T antigen. We selected HEK293T cells because these were p53 mutated cells that exhibited transformed growth on soft agar, showed tumorigenic potential (form tumors in nude mice) and had high transfection efficacy.^25^ Consistent with previous results, there was a significant reduction in GFP fluorescence intensity in ultrasound‐treated tumors. In contrast, non‐treated tumors showed increased intensity, indicating growth (Figure S8). Thus, the ultrasound mechanosensitivity was not limited to a specific tumor or tumor cell line.

### 2.5 Ultrasound causes apoptosis of patient‐derived tumor organoids

**FIGURE 5:**
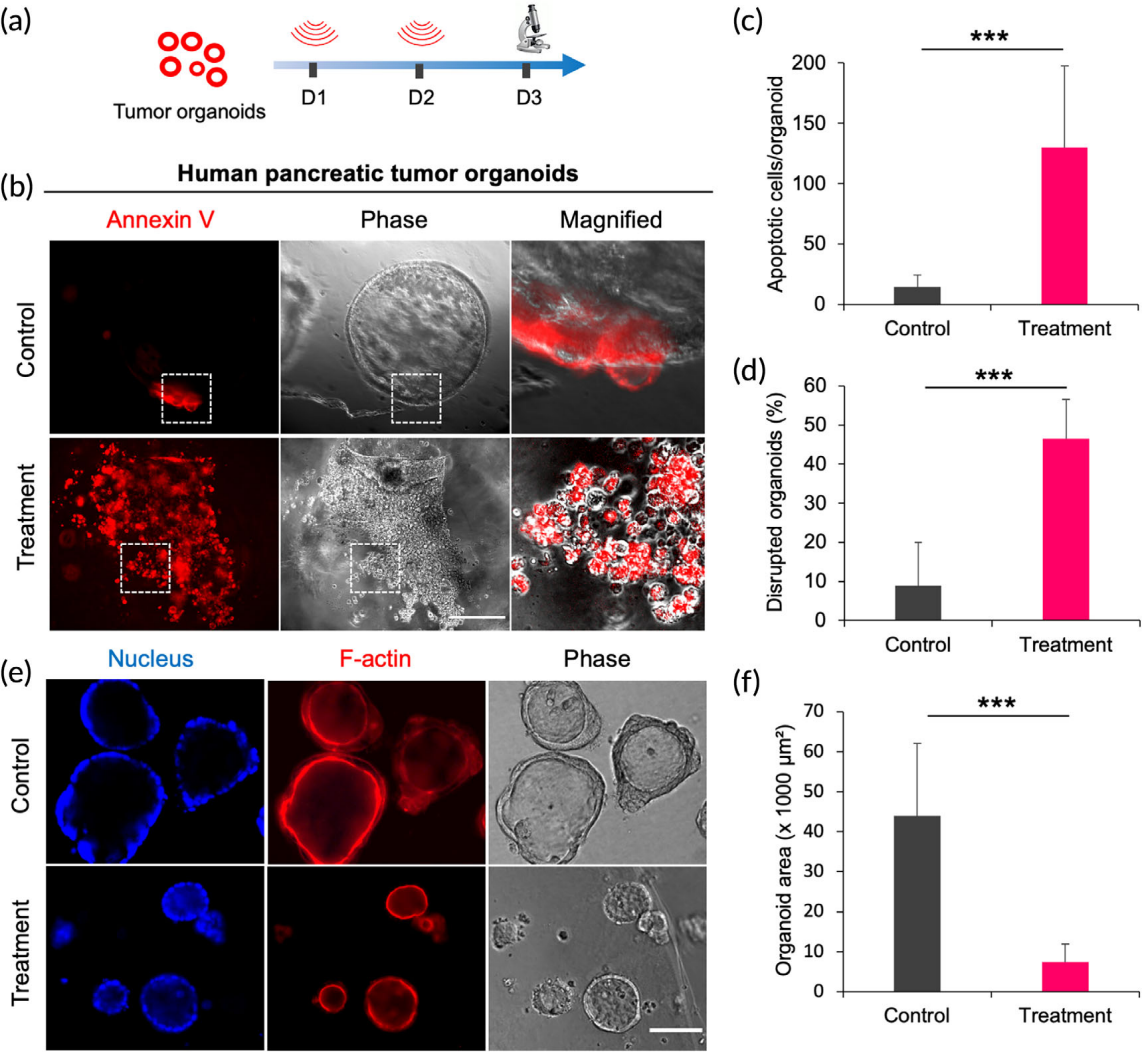
Ultrasound (33 kHz) promotes apoptosis in human pancreatic tumor organoids. (a) Timeline depicting the scheme of tumor organoid US treatment. (b) Representative images showing apoptotic cells/organoids in control and treated samples after two rounds of ultrasound, scale bar: 100 μm. (c) Bar diagram showing the extent of apoptosis per organoids in the control and treated organoids. *n* > 25 organoids, Student's *t*‐test, ***p < 0.001. (d) Bar diagram estimating the disrupted organoid population in the control and treated organoids. *n* > 100 organoids, Student's *t*‐test, ***p < 0.001. (e) Immunofluorescence staining of F‐actin and nucleus of organoids in control and treated samples, scale bar: 100 μm. (f) Bar diagram showing the organoid area in the control and treated organoids. *n* > 100 organoids, Student's *t*‐test, ***p < 0.001.

To further validate the ultrasound‐induced mechanoptosis, human pancreatic tumor organoids were subjected to ultrasound treatment. Organoids were derived from primary tissue/tumor biopsies. They were composed of organ‐specific cell‐type aggregates capable of self‐renewal, self‐organization and retained organ functionality.^25^, ^26^ Freshly prepared organoids were treated with ultrasound (33 kHz) for 2 h/day for two consecutive days, having a power level of 39 mW/cm^2^ (Figure 5a). On Day 3, there was a marked increase in the apoptosis level in treated tumor organoids compared to the controls (Figure 5b,c). In addition, many organoids were disrupted and had a broken and irregular morphology in the treated samples (Figures 5d and S8). These disrupted organoids were surrounded by many isolated cells/aggregates, which exhibited a high level of apoptosis. The remaining organoids in the treated sample were significantly smaller (~7500 μm^2^) when compared with the controls (~44,000 μm^2^) (Figures 5f and S9). It seems that disrupted organoids reorganized to form smaller organoids. Next, F‐actin imaging revealed no visible difference in stress fiber assembly in the treated and control groups (Figure 5e). Thus, the results illustrated that the human‐derived tumor cells in an organoid that approximated the organization of in vivo tumors were vulnerable to ultrasound‐mediated mechanical forces like the established tumor cell lines.

### 2.6 Ultrasound causes tumor regression in a mouse model

**FIGURE 6:**
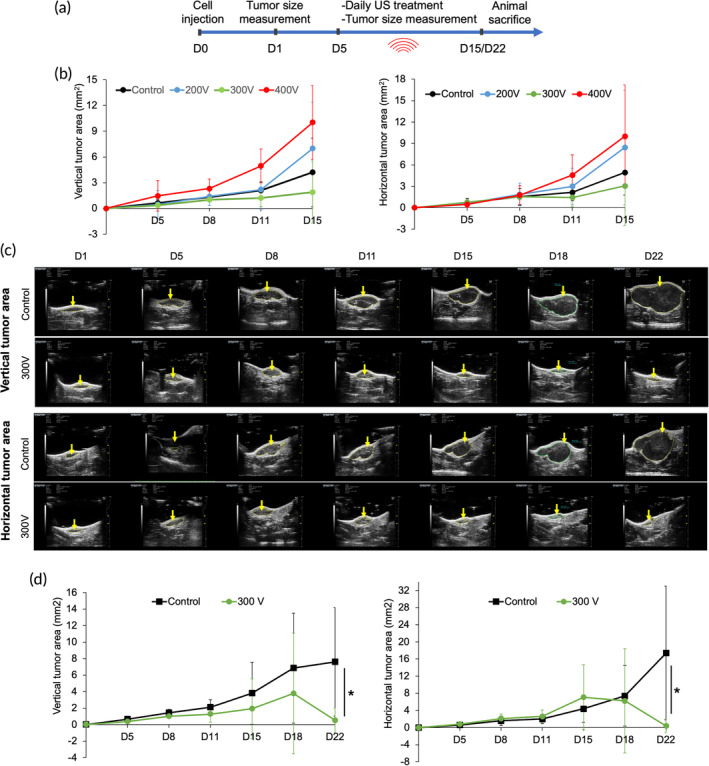
Ultrasound (39 kHz) inhibits tumor growth in a mouse model. (a) Timeline depicting the scheme of in vivo tumor treatment using ultrasound. (b) The graph depicting the vertical and horizontal tumor area after treatment with different US power levels of 200, 300, and 400 V for 2 weeks. *n* = 5 for control, *n* = 6 for 200 V, *n* = 4 for 300 V, and *n* = 5 for 400 V. (c) The representative ultrasound scanned images of tumor areas in untreated and treated (300 V) mouse models at different time points. The yellow arrow denotes the tumor region. (d) The graph showing the vertical and horizontal tumor area in untreated and treated (300 V) mouse models until day 22. *n* = 5 for control, and *n* = 4 for 300 V, Student's *t*‐test, *p < 0.05.

We tested the efficiency of low‐frequency ultrasound in treating in vivo tumors using a mouse model. As the abovementioned findings suggested that the ultrasound power levels significantly affected the apoptosis levels (Figure S4), animals were treated at 39 kHz ultrasound frequency for 1 h/day for 2–3 weeks using different power levels 39 mW/cm^2^ (200 V), 51 mW/cm^2^ (300 V), and 166 mW/cm^2^ (400 V) (Figure 6a,b). Surprisingly, the lowest tumor growth was observed in the group treated with 300 V (2–3 mm^2^) compared to the 200 V (7–8.5 mm^2^), 400 V (~10 mm^2^), and control groups (4–5 mm^2^) after 2 weeks. However, tumor growth inhibition varied greatly within each treated group and was evident in notable variations during tumor area measurement.

Since mice treated with 300 V showed the lowest tumor growth, we further tested the effect of 300 V power level on tumor growth for a more extended period. Interestingly, 300 V treatment caused a significant reduction in tumor growth area (~1 mm^2^) after 22 days of treatment compared to the untreated control group (7–17 mm^2^) (Figure 6c,d). These findings suggested that low‐frequency ultrasound treatment with optimized parameters can inhibit in vivo tumor growth. Also, these results open up the possibility of combining optimized ultrasound treatment with existing therapies to augment cancer treatment.

## 3 DISCUSSION

This study finds that ultrasound‐mediated mechanical forces kill tumor cells under conditions that do not damage normal cells/tissues. The tissue origin of the cells does not significantly affect the level of apoptosis caused by ultrasound treatment. Apoptosis requires the presence of Piezo1 at the cell periphery and appears to be mediated by mechanically‐dependent calcium entry that activates calpain to cause an apoptotic cascade. The effects of ultrasound on chick CAM tumors, patient‐derived tumor organoids and a tumor mouse model are dramatic, with significant killing of tumor cells in physiologically relevant tissue forms and in vivo. Thus, we suggest that ultrasound acts at a subcellular level that is relatively insensitive to the cell microenvironment but depends upon the transformed state of the tumor cells.^9^ Repetitive ultrasound treatment caused a substantial reduction in several in vitro models and animal studies, indicating that ultrasound treatment did not develop mechanically primed resistant cells in the time frame we used. In‐depth in vivo studies are required to check the development of mechanically primed resistant cells and their role in metastasis upon ultrasound treatment. The fact that the higher powers did cause increased tumor growth indicates that the range power level for the killing of tumor cells is relatively narrow.

This selective killing correlates with differences in the mechanical properties of tumors and normal cells.^19^ For example, all tumor cell lines we have tested lack rigidity sensors (e.g., tropomyosin 2.1 and myosin IIA), enabling them to grow on soft surfaces while making them sensitive to apoptosis upon cyclic stretching.^6^, ^8^, ^10^ In contrast, normal cells with rigidity sensors show apoptosis on soft surfaces but are not killed by cyclic stretching.^7^ Interestingly, restoration of rigidity sensors in tumor cells promotes rigidity‐dependent growth, improves cell stiffness, and even grows when subjected to cyclic stretching.^6^, ^8^ In contrast, the depletion of rigidity sensors in normal cells initiates transformed growth and apoptosis upon cyclic stretching. Since we have tested cells from different tissues, these findings indicate that tumor cells from many tissues are vulnerable to mechanical forces irrespective of their microenvironment. In contrast, normal cells withstand mechanical treatments and continue to grow in a proper environment.^1^, ^5^, ^27^

In the past, several attempts have been made to cause tumor cell killing with ultrasound. However, those were based on either micro/nanobubble‐generated thermal cavitation, thermal ablation or focused high‐energy waves, often associated with a high risk of local healthy tissue damage due to overheating.^28^ For instance, high‐intensity focused ultrasound (HIFU) has been used clinically to ablate tumors located within the body, but its application is limited due to severe thermal side effects and limited ability to treat metastatic tumors.^29^, ^30^ In stark contrast, ultrasound‐mediated mechanical forces used in these studies did not cause temperature to rise above 37°C during in vitro or in vivo conditions. To minimize thermal heating, degassed water was used in the tank to remove water microbubbles and prevent thermal heat generated by the microbubble cavitation effect.^31^ In addition, a 50% duty cycle was used during the treatment to ensure the system was ON for 1 s and OFF for 1 s. This periodic ON–OFF functioning provides a cool‐off time and prevents excessive heat generation. Hence, after optimized ultrasound treatment, normal cells (MCF10A and HFF) remain unharmed (Figures 1 and 2). More importantly, in the CAM model, tumor cells were killed with a lower risk to the chick embryo, which is highly vulnerable to ultrasound treatment (Figure 5f).^32^ Similarly, tumor growth was effectively inhibited in mice with ultrasound treatment without causing significant damage to animals (Figure 6). Thus, optimized ultrasound‐mediated mechanical forces preferentially promote tumor cell apoptosis in vitro and in vivo (Table S1). However, further studies are required to optimize the ultrasound parameters to maximize the selective killing of tumor cells in vivo and examine the combined effect of ultrasound treatment and chemotherapeutic drugs on tumor suppression.

## 4 CONCLUSION

**FIGURE 7:**
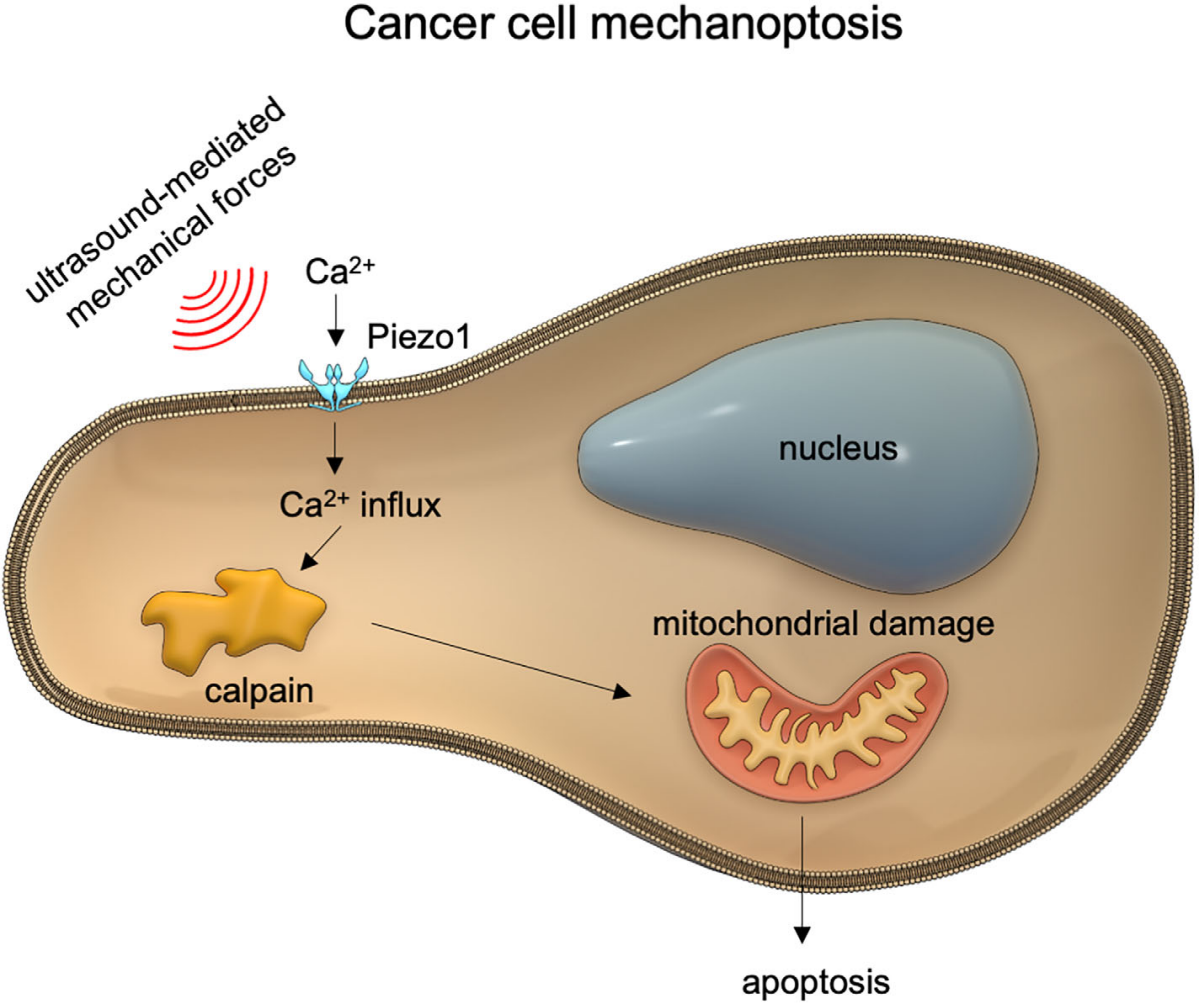
Schematic illustrating the mechanism of ultrasound‐mediated tumor cell mechanoptosis. Ultrasound‐generated mechanical forces activate mechanosensitive Piezo1 channels to initiate rapid calcium entry. Calcium‐activated calpain then triggers the mitochondrial apoptotic pathway through chain reactions to induce mechanoptosis.

In summary, we showed that ultrasound‐mediated mechanical forces caused selective killing of tumor cells in vivo and in vitro without significant damage to normal cells. At a mechanistic level, ultrasound‐induced mechanoptosis appears to act similarly to mechanical stretch‐induced mechanoptosis.^6^ The ultrasound‐mediated forces activate Piezo1 channels that allow calcium entry, activating calpain and subsequently initiating a mitochondrial apoptotic pathway (Figure 7). This calcium‐induced apoptosis is similar to calcium‐dependent apoptosis of tumor cells through the ER‐mitochondrial stress pathway activated by several natural compounds.^33^, ^34^ With a more detailed understanding of the mechanism of tumor cell death, many possible cancer therapies would enhance the effect of low‐frequency ultrasound treatment. Because ultrasound has been approved for human exposure at power levels 10–100‐fold higher than the levels used in this study,^28^ we suggest that it is practical to develop mechano‐based therapies using ultrasound that could augment tumor treatments.

## 5 MATERIALS AND METHODS

### 5.1 Cell culture

Cell lines MDA‐MB‐231 (gift from Dr. Jay Groves, UC Berkeley), A375p (gift from Dr. Chris Bakal, ICR UK) and primary human foreskin fibroblasts (HFF, ATCC), HT1080 (ATCC), and SKOV3 (gift from Dr. Ruby Huang, CSI, NUS) were cultured in high glucose DMEM (ThermoFisher) supplemented with 10% FBS and 1% penicillin and streptomycin at 37°C in 5% CO_2_ environment. MCF10A, human breast epithelial cells (ATCC) were grown in a specialized culture medium, as mentioned previously.^7^ For cell experiments, high‐glucose DMEM containing 10% FBS and 1% penicillin and streptomycin were utilized. TrypLE (ThermoFisher) was used for cell trypsinization and cells were cultured on matrigel (Corning) coated surfaces.

### 5.2 Surface coating with matrigel

The 96‐well plate surface was coated with a thin matrigel (Corning) layer per the manufacturer's protocol. Briefly, after thawing matrigel at 4°C, 33 μL of matrigel solution was quickly added onto the pre‐chilled surface of a single well of the 96‐well plate. The well plates were incubated at 37°C for 24–48 h to solidify matrigel before performing a cell seeding.

### 5.3 Plasmids and transfection

GFP tagged‐stable MDA‐MB‐231 cell line was a gift from Pan Meng (MBI, NUS) and used for in vivo study. For transient transfection of GFP and mApple‐paxillin plasmid, Lipofectamine 3000 reagent (ThermoFisher) was used according to the manufacturer's protocol.

### 5.4 siRNA assay

Cells were plated in a six‐well plate to perform assays. Calpain1, calpain2, Piezo1, and scramble siRNA (Sigma) transfections were done the next day using lipofectamine RNAiMAX (Invitrogen) according to the manufacturer's protocol. Calpain siRNAs were prepared by Protein Cloning Expression Facility, MBI, NUS as described previously.^6^

### 5.5 Western blot study

The cells were incubated with siRNA for 48 h, pelleted, and washed with PBS. RIPA buffer (Sigma) supplemented with 1× complete protease inhibitor (Roche, No. 4693116001) was used for cell lysis. The cell mixture was centrifuged at 15,000 rpm, 4°C for 20 min. The supernatant was transferred to Eppendorf and mixed with 2× loading dye (2× Lammeli buffer, Bio‐Rad, No. 1610737) + beta‐Mercaptoethanol (Sigma). Samples were denatured for 5 min at 95°C and gel was run using 4%–20% Mini‐PROTEAN® TGX precast protein gels (Bio‐Rad, No. 4561094). Next, gels were then transferred onto blot paper and blocked with 5% BSA solution in 1× TBST (Tris‐Buffered Saline with Tween‐20) for 1 h. The membrane was then incubated with primary antibody overnight at 4°C on a shaker. Membranes were washed with TBST three times (10 min per wash) and incubated with secondary antibodies in 1× TBST (horse radish peroxidase, HRP) for 1 h. The chemiluminescence of the membranes was developed using Super Signal Femto Substrate Kit (Pierce) and developed using ChemiDoc Touch Imager (Bio‐Rad). Primary antibodies and conditions used are as follows: Calpain 1 (rabbit, 1:1000, Abcam, ab39170), Calpain 2 (rabbit, 1:1000, Abcam, ab39165), Piezo1 (rabbit, 1:500, Novus Biologicals, NBP1‐78446), and alpha‐tubulin (mouse, 1:3000, Sigma T9026). The secondary antibodies HRP–conjugated goat anti‐rabbit IgG (1:2000, Bio‐Rad, 170‐6515) and goat anti‐mouse IgG (1:2000, Bio‐Rad, 170‐6516).

### 5.6 Apoptosis and necrosis assay

To identify cell apoptosis, Annexin V‐Alexa Fluor 488 or Annexin V‐Alexa Fluor 594 conjugates (Thermofisher Scientific) were used according to the manufacturer's protocol. To check cell necrosis, propidium iodide from a live/dead cell double staining kit (Sigma Aldrich) was used according to the manufacturer's protocol. Assay was performed 12 h after ultrasound treatment.

### 5.7 Live cell assay

Calcein AM (Sigma Aldrich) dye was used to check the cell viability according to the manufacturer's protocol. Cells were incubated with dye every day for 15 min before imaging.

### 5.8 Calcium indicator dye‐based assay

Cell samples were incubated with the calcium indicator dye (4 mM of Cal‐520AM, AAT Bioquest) for 1 h. Samples were then replenished with fresh culture medium and stabilized for 30 min before ultrasound treatment.

### 5.9 Immunocytochemistry and fluorescence microscopy

Samples were fixed with 4% paraformaldehyde (Thermofisher Scientific) solution for 10 min and permeabilized with 0.2% Triton X‐100 for 5 min. Normal goat serum (2%) was used as a blocking buffer and samples were treated with serum for 1 h. Samples were then incubated with primary antibodies of rabbit polyclonal anti‐Piezo1 (1:200, Novus Biologicals, catalog no. NBP1‐78446) overnight at 4°C followed by treatment with Alexa Fluor‐594 secondary antibodies (Thermofisher Scientific). Hoechst dye (1:1000, Thermofisher Scientific) was used to stain the cell nucleus.

For in vitro study, fluorescence and bright field images were acquired using a wide‐field Olympus live‐EZ microscope equipped with a photometric CoolSNAP K4 camera and W1 live‐SR spinning disk microscope equipped with a photometric Prime 95B sCMOS camera. For in vivo tumor imaging, a wide‐field Zeiss stereomicroscope was used.

### 5.10 In vitro ultrasound treatment

The custom‐made ultrasound device was developed in the lab for ultrasound treatment (Figure S10) as mentioned previously.^35^ Briefly, we used ring transducers with diameters of 16 and 25 mm made of PZT4 material (Beijing Ultrasonics 25 × 10 × 4 and 16 × 8 × 4 piezoceramic rings).^36^ An aluminum cone is mounted on the rings to widen the field to some 5 cm diameter and attenuate the amplitude to form plane waves. Epoxy and silicon rubberized coats were added to transducers to improve the wave emission. The transducer was fixed to the bottom of the ultrasound water tanks. The water tank was placed in an incubator to maintain 37°C and moisture. The cell well plates/dishes were sealed with parafilm to avoid water entry inside samples during US treatment. Sealed samples were then placed 8 cm above the transducer top. The tank was filled with degassed DI water to immerse the cell samples partially. All primary signals and sequences were generated by Arduino's microcontrollers. This allowed for the swift alteration of duty cycles, sequences, and duration. Cell samples were treated with 33 or 120 kHz frequency with a 50% duty cycle for 2 h. The cell samples were returned to the incubator for overnight incubation to determine the apoptosis level on the following day. The speed of ultrasound in soft tissues is considered as 1540 m/s. Hence, the ultrasound wavelengths at 33 and 120 kHz frequency are 4.6 and 1.2 cm, respectively. The penetration depth for both frequencies is up to 6 cm depending on tissue complexity.

### 5.11 Tumor organoid formation

Pancreatic tumor tissue was obtained from patients undergoing endoscopic surgical resection or tissue biopsy at National University Hospital, Singapore. All tissue donations and experiments were reviewed and approved by the Institutional Review Board. Tissue samples were collected in Hank's Balanced Salt Solution and transferred from the hospital at 4°C. Tissues were minced and plated in Matrigel (Corning). The cell culture medium used is as follows: advanced DMEM/F12, HEPES 10 mM, Glutamax 1×, A8301 500 nM, hEGF 50 ng/mL, mNoggin 100 ng/mL, hFGF10 100 ng/mL, hGastrin I 0.01 μM, N‐acetylcysteine 1.25 mM, Nicotinamide 10 mM, PGE2 1 μM, B27 supplement 1× final, R‐spondin1, and Wnt3A.

### 5.12 CAM tumor treatment with ultrasound

Fertilized chicken eggs were incubated at 37°C with 70% humidity starting from embryonic day 0 (ED 0). A window was made on eggs on ED 3 to prevent the CAM from sticking onto the shell during development. Under sterile conditions, a small incision was made at the pointed end of the egg using a sharp surgical rod or needle, and 4–5 mL of albumin was removed through the hole to lower the CAM. The eggshell was then cut to form an approximate 1 cm diameter window, exposing the CAM. This window was sealed with Tegaderm transparent film dressing (3 M). Eggs were further incubated to ED7 until they were ready for tumor grafting. GFP‐transfected stable MDA‐MB‐231 and GFP‐transfected (transiently expressed) transformed HEK293T cells were used for grafting purposes. For grafting onto CAM, tumor cells were trypsinized, washed by PBS, and re‐suspended in pre‐chilled Matrigel (Corning). Next, the eggshell was further cut to form a larger window. A slightly bruised area was developed by gently touching the upper periderm layer of the CAM with an autoclaved glass rod, and one million cells in 50 μL Matrigel were inoculated drop‐wise onto the bruised area. After inoculation, the window was sealed again with a film dressing. On ED10, tumors formed were imaged using a stereomicroscope (optical and fluorescence) before being treated with an ultrasound. Fertilized eggs containing tumors were subjected to ultrasound for 2 h. The procedure was done for two consecutive days, ED10 and ED11. Tumor imaging was again performed on ED12 to compare the tumor growth. Only tumors with viable embryos were included in the data analysis.

### 5.13 Animal studies

A 7 months old BALB/cJ mice were used for the in vivo experiments. Animal experiments were done as per IACUC guidelines of UTMB, Galveston (protocol number 1910084A). Subcutaneous injections of 50,000 cells of CT26 (murine colorectal carcinoma) mixed with 80% Matrigel were injected in each mouse. The baseline tumor area was measured by performing an ultrasound scan the next day (Day 1) after the injecting cells. Mice were placed in a chamber filled with degassed water on a 1.5 cm thick gel pad directly placed on the transducer. The ultrasound treatment was initiated from Day 5 post‐injection and given every day for 2 weeks. Animals were treated at 39 kHz for 1 h at different ultrasound power levels of 200, 300, and 400 V. To monitor the tumor growth, ultrasound scans of the tumor's vertical and horizontal cross‐sectional area were captured every 3 days. The change in tumor size was calculated using the following formula.

Change in tumor size = [tumor size on Day X – tumor size on Day 1]/tumor size on Day 1.

## AUTHOR CONTRIBUTIONS

A.T., F.M., and M.S. conceived and designed the project; A.T. performed cell and organoid experiments; F.M. fabricated ultrasound devices; A.T. and A.H. performed CAM experiments; A.T., M.Y., and S.P. performed calcium‐related experiments; N.D. performed animal experiments; C.A.Z.C. and G.B. developed tumor organoids; A.T., F.M., and M.S. wrote the manuscript. All authors read and commented on the manuscript.

## CONFLICT OF INTEREST STATEMENT

A.T., F.M., M.Y. and M.S. are inventors of patent applications related to the technology described in this paper. F.M. and M.S. have formed a company, Mechanobiologics Inc., to develop ultrasound therapies for human cancer treatment.

## ETHICS STATEMENT

Animal experiments were done as per IACUC guidelines of UTMB, Galveston (protocol number 1910084A). Humane care and treatment of animals were ensured.

## Supporting information


**APPENDIX S1:** Supplementary information.

**VIDEO S1:** MDA‐MB‐231 cells loaded with calcium‐sensitive dye Cal‐520 AM showing changes in dye intensity with US treatment.

**VIDEO S2:** MCF10A cells loaded with calcium‐sensitive dye Cal‐520AM showing changes in dye intensity with US treatment.

## Data Availability

The data that support the findings of this study are available from the corresponding author upon reasonable request.

## References

[1] Berrueta L , Bergholz J , Munoz D , et al. Stretching reduces tumor growth in a mouse breast cancer model. Sci Rep. 2018;8(1):7864. 29777149 10.1038/s41598-018-26198-7 PMC5959865

[2] Betof AS , Lascola CD , Weitzel D , et al. Modulation of murine breast tumor vascularity, hypoxia and chemotherapeutic response by exercise. J Natl Cancer Inst. 2015;107(5):djv040. 25780062 10.1093/jnci/djv040 PMC4822524

[3] Bray F , Ferlay J , Soerjomataram I , Siegel RL , Torre LA , Jemal A . Global cancer statistics 2018: GLOBOCAN estimates of incidence and mortality worldwide for 36 cancers in 185 countries. CA Cancer J Clin. 2018;68(6):394‐424. 30207593 10.3322/caac.21492

[4] Lien S‐C , Chang S‐F , Lee P‐L , et al. Mechanical regulation of cancer cell apoptosis and autophagy: roles of bone morphogenetic protein receptor, Smad1/5, and p38 MAPK. Biochim Biophys Acta. 2013;1833(12):3124‐3133. 24021264 10.1016/j.bbamcr.2013.08.023

[5] Regmi S , Fu A , Luo KQ . High shear stresses under exercise condition destroy circulating tumor cells in a microfluidic system. Sci Rep. 2017;7:39975. 28054593 10.1038/srep39975 PMC5215453

[6] Tijore A , Yao M , Wang Y‐H , et al. Selective killing of transformed cells by mechanical stretch. Biomaterials. 2021;275:120866. 34044258 10.1016/j.biomaterials.2021.120866

[7] Wolfenson H , Meacci G , Liu S , et al. Tropomyosin controls sarcomere‐like contractions for rigidity sensing and suppressing growth on soft matrices. Nat Cell Biol. 2016;18(1):33‐42. 26619148 10.1038/ncb3277 PMC5296190

[8] Yang B , Wolfenson H , Chung VY , et al. Stopping transformed cancer cell growth by rigidity sensing. Nat Mater. 2020;19(2):239‐250. 31659296 10.1038/s41563-019-0507-0 PMC7477912

[9] Sheetz M . A tale of two states: Normal and transformed, with and without rigidity sensing. Annu Rev Cell Dev Biol. 2019;35:169‐190. 31412209 10.1146/annurev-cellbio-100818-125227 PMC7474971

[10] Tijore A , Yang B , Sheetz M . Cancer cells can be killed mechanically or with combinations of cytoskeletal inhibitors. Front Pharmacol. 2022;13:955595. 36299893 10.3389/fphar.2022.955595 PMC9589226

[11] Wood AKW , Sehgal CM . A review of low‐intensity ultrasound for cancer therapy. Ultrasound Med Biol. 2015;41(4):905‐928. 25728459 10.1016/j.ultrasmedbio.2014.11.019 PMC4362523

[12] Rapoport NY , Kennedy AM , Shea JE , Scaife CL , Nam K‐H . Controlled and targeted tumor chemotherapy by ultrasound‐activated nanoemulsions/microbubbles. J Controll Release: Off J Controll Release Soc. 2009;138(3):268‐276. 10.1016/j.jconrel.2009.05.026 PMC2746980 19477208

[13] Feril LB Jr , Kondo T , Zhao QL , Ogawa R . Enhancement of hyperthermia‐induced apoptosis by non‐thermal effects of ultrasound. Cancer Lett. 2002;178(1):63‐70. 11849742 10.1016/s0304-3835(01)00826-6

[14] Guan L , Xu G . Damage effect of high‐intensity focused ultrasound on breast cancer tissues and their vascularities. World J Surg Oncol. 2016;14(1):153. 27230124 10.1186/s12957-016-0908-3 PMC4882851

[15] Ashush H , Rozenszajn LA , Blass M , et al. Apoptosis induction of human myeloid leukemic cells by ultrasound exposure. Cancer Res. 2000;60(4):1014‐1020. 10706118

[16] Mittelstein DR , Ye J , Schibber EF , et al. Selective ablation of cancer cells with low intensity pulsed ultrasound. Appl Phys Lett. 2020;116(1):013701.

[17] Rubin MD , Anderton N , Smalberger C , Polliack J , Nathan M , Postema M . On the behaviour of living cells under the influence of ultrasound. Fluids. 2018;3(4):82.

[18] Ahmadi F , McLoughlin IV , Chauhan S , ter‐Haar G . Bio‐effects and safety of low‐intensity, low‐frequency ultrasonic exposure. Prog Biophys Mol Biol. 2012;108(3):119‐138. 22402278 10.1016/j.pbiomolbio.2012.01.004

[19] Lejbkowicz F , Salzberg S . Distinct sensitivity of normal and malignant cells to ultrasound in vitro. Environ Health Perspect. 1997;105(suppl 6):1575‐1578. 9467085 10.1289/ehp.97105s61575 PMC1469937

[20] Klibanov AL , Hossack JA . Ultrasound in radiology: from anatomic, functional, molecular imaging to drug delivery and image‐guided therapy. Invest Radiol. 2015;50(9):657‐670. 26200224 10.1097/RLI.0000000000000188 PMC4580624

[21] Kumari A , Veena SM , Luha R , Tijore A . Mechanobiological strategies to augment cancer treatment. ACS Omega. 2023;8(45):42072‐42085. 38024751 10.1021/acsomega.3c06451 PMC10652740

[22] Coste B , Mathur J , Schmidt M , et al. Piezo1 and Piezo2 are essential components of distinct mechanically activated cation channels. Science (New York, NY). 2010;330(6000):55‐60. 10.1126/science.1193270 PMC3062430 20813920

[23] Chen X , Wanggou S , Bodalia A , et al. A feedforward mechanism mediated by mechanosensitive Ion Channel PIEZO1 and tissue mechanics promotes glioma aggression. Neuron. 2018;100(4):799‐815. 30344046 10.1016/j.neuron.2018.09.046

[24] Yao M , Tijore A , Cheng D , et al. Force‐ and cell state‐dependent recruitment of Piezo1 drives focal adhesion dynamics and calcium entry. Sci Adv. 2022;8(45):eabo1461. 36351022 10.1126/sciadv.abo1461 PMC9645726

[25] Stepanenko AA , Dmitrenko VV . HEK293 in cell biology and cancer research: phenotype, karyotype, tumorigenicity, and stress‐induced genome‐phenotype evolution. Gene. 2015;569(2):182‐190. 26026906 10.1016/j.gene.2015.05.065

[26] Clevers H . Modeling development and disease with organoids. Cell. 2016;165(7):1586‐1597. 27315476 10.1016/j.cell.2016.05.082

[27] Cui Y , Hameed FM , Yang B , et al. Cyclic stretching of soft substrates induces spreading and growth. Nat Commun. 2015;6(1):6333. 25704457 10.1038/ncomms7333 PMC4346610

[28] Shankar H , Pagel Paul S . Potential adverse ultrasound‐related biological effects: a critical review. Anesthesiol: J Am Soc Anesthesiol. 2011;115(5):1109‐1124. 10.1097/ALN.0b013e31822fd1f1 21866043

[29] Zhou Y‐F . High intensity focused ultrasound in clinical tumor ablation. World J Clin Oncol. 2011;2(1):8‐27. 21603311 10.5306/wjco.v2.i1.8 PMC3095464

[30] Miller DL , Smith NB , Bailey MR , et al. Overview of therapeutic ultrasound applications and safety considerations. J Ultrasound Med. 2012;31(4):623‐634. 22441920 10.7863/jum.2012.31.4.623 PMC3810427

[31] Zhao Z , Saiding Q , Cai Z , Cai M , Cui W . Ultrasound technology and biomaterials for precise drug therapy. Mater Today. 2023;63:210‐238.

[32] Dixon FJ . The effects of radiation on the chick embryo. Ann N Y Acad Sci. 1952;55(2):216‐220. 12977038 10.1111/j.1749-6632.1952.tb26537.x

[33] Kaufman RJ , Malhotra JD . Calcium trafficking integrates endoplasmic reticulum function with mitochondrial bioenergetics. Biochim Biophys Acta (BBA)—Mol Cell Res. 2014;1843(10):2233‐2239. 10.1016/j.bbamcr.2014.03.022 PMC4285153 24690484

[34] Kim C , Kim B . Anti‐cancer natural products and their bioactive compounds inducing ER stress‐mediated apoptosis: a review. Nutrients. 2018;10(8):1021. 30081573 10.3390/nu10081021 PMC6115829

[35] Tijore AS , Margadant F , Yao M , Sheetz MP . Systems and methods for cancer treatment: US patent app 17/607,819. 2022.

[36] DeAngelis DA , Schulze GW . Performance of PZT8 versus PZT4 piezoceramic materials in ultrasonic transducers. Phys Proc. 2016;87:85‐92.

