# Supplementary material for: Ultrasound‐mediated mechanical forces activate selective tumor cell apoptosis
Source: Bioeng Transl Med. 2024 Dec 30;10(2):e10737. doi: 10.1002/btm2.10737 (PMC11883105; doi:10.1002/btm2.10737)
Supplement: Supplementary file 1 — APPENDIX S1: Supplementary information. [file BTM2-10-e10737-s002.pdf]

Supporting Information

Ultrasound-mediated mechanical forces activate selective tumor cell apoptosis

Ajay Tijore[1,2,†] Felix Margadant[1,3,†] Nehal Dwivedi[3], Leslie Morgan[3], Mingxi Yao[1], Anushya Hariharan[1], Claire Alexandra Zhen Chew[4], Simon Powell[3], Glenn Kunnath Bonney[4], Michael Sheetz[1,3]*

[1]Mechanobiology Institute, National University of Singapore, Singapore

[2]Department of Bioengineering, Indian Institute of Science, Bangalore, Karnataka, India

[3]Biochemistry and Molecular Biology Department, University of Texas Medical Branch, Galveston, Texas, USA

[4]Division of Hepatobiliary & Pancreatic Surgery, Department of Surgery, National University Hospital, Singapore

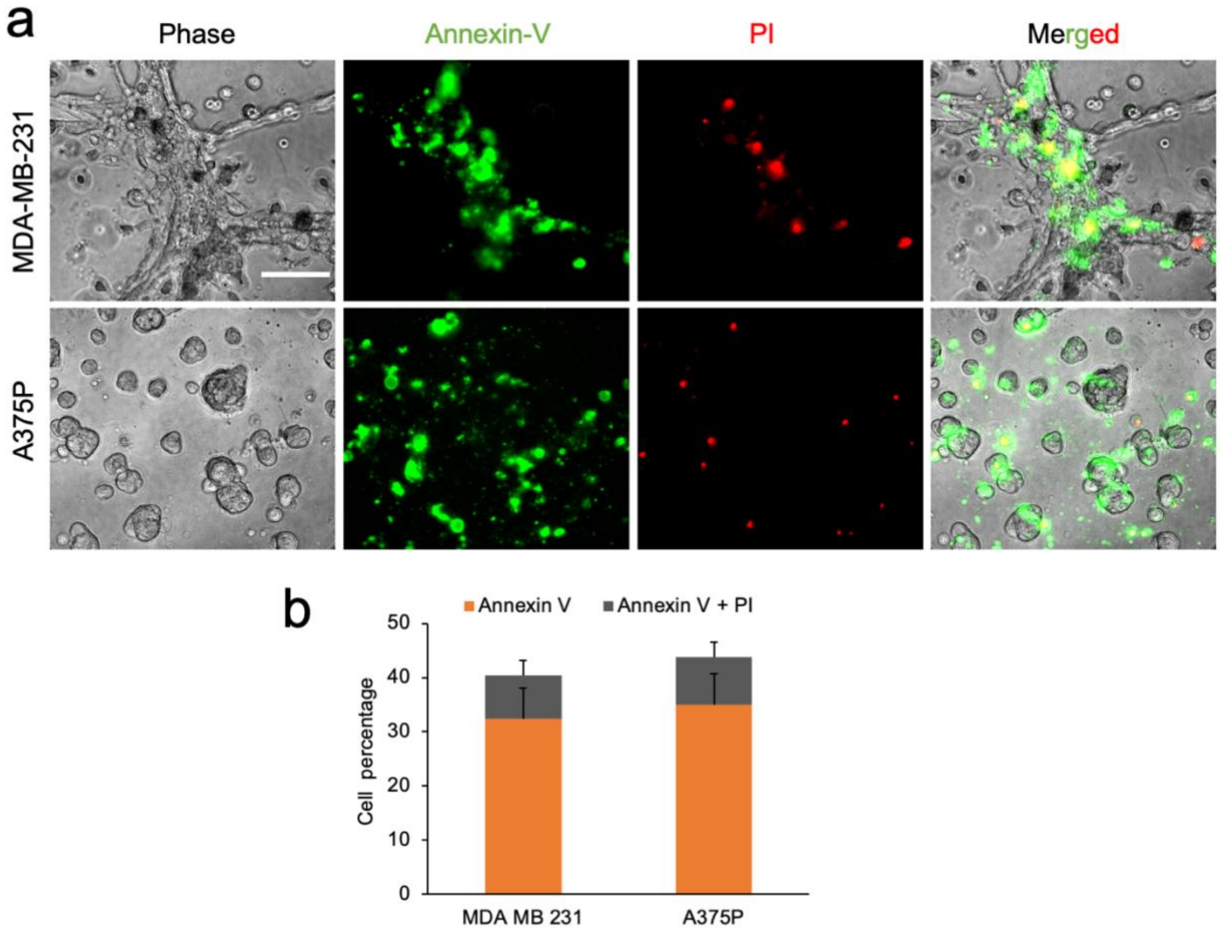

Figure S1. Ultrasound (33 kHz) promotes apoptosis in tumor cells (a) Representative images displaying apoptotic (annexin V) and necrotic (propidium iodide) cells after the treatment, scale 100 µm. (b) Bar diagram demonstrating the percentage of cells showing apoptosis and necrosis with treatment, n>650 cells.

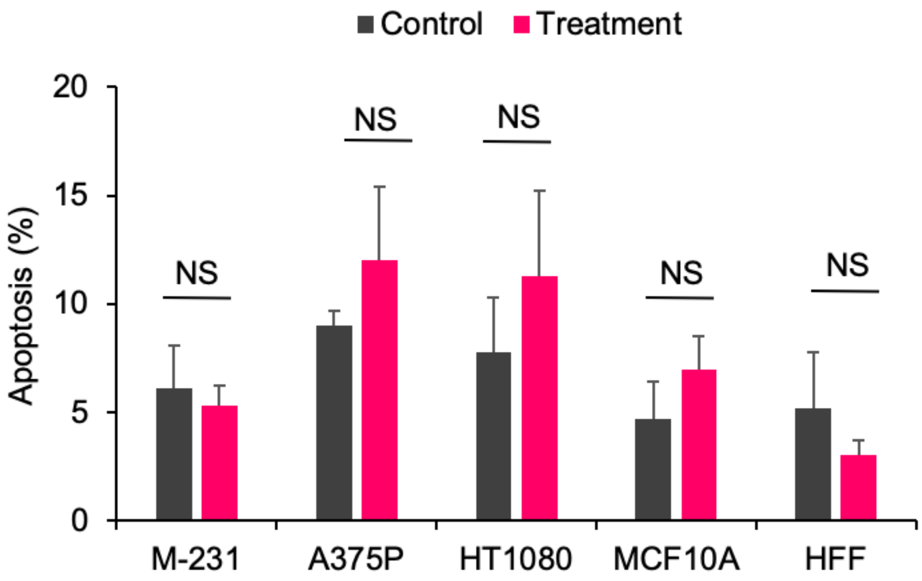

Figure S2. Ultrasound at 120 kHz frequency does not cause tumor cell apoptosis. Bar diagram illustrating apoptosis level in tumor (MDA-MB-231, A375p, HT1080) and normal cells (MCF10A, HFF) with and without ultrasound treatment. n>1000 cells, data represent two independent experiments, ANOVA test, p <0.05, no significance.

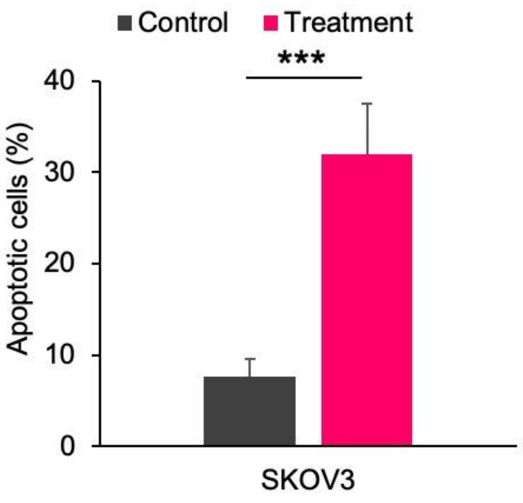

Figure S3. Ultrasound (33 kHz) induces apoptosis in tumor cells from different tissue origins. Bar diagram showing apoptosis level in SKOV3, ovarian adenocarcinoma with and without ultrasound treatment. n>750 cells, student t-test, *p****< 0.001

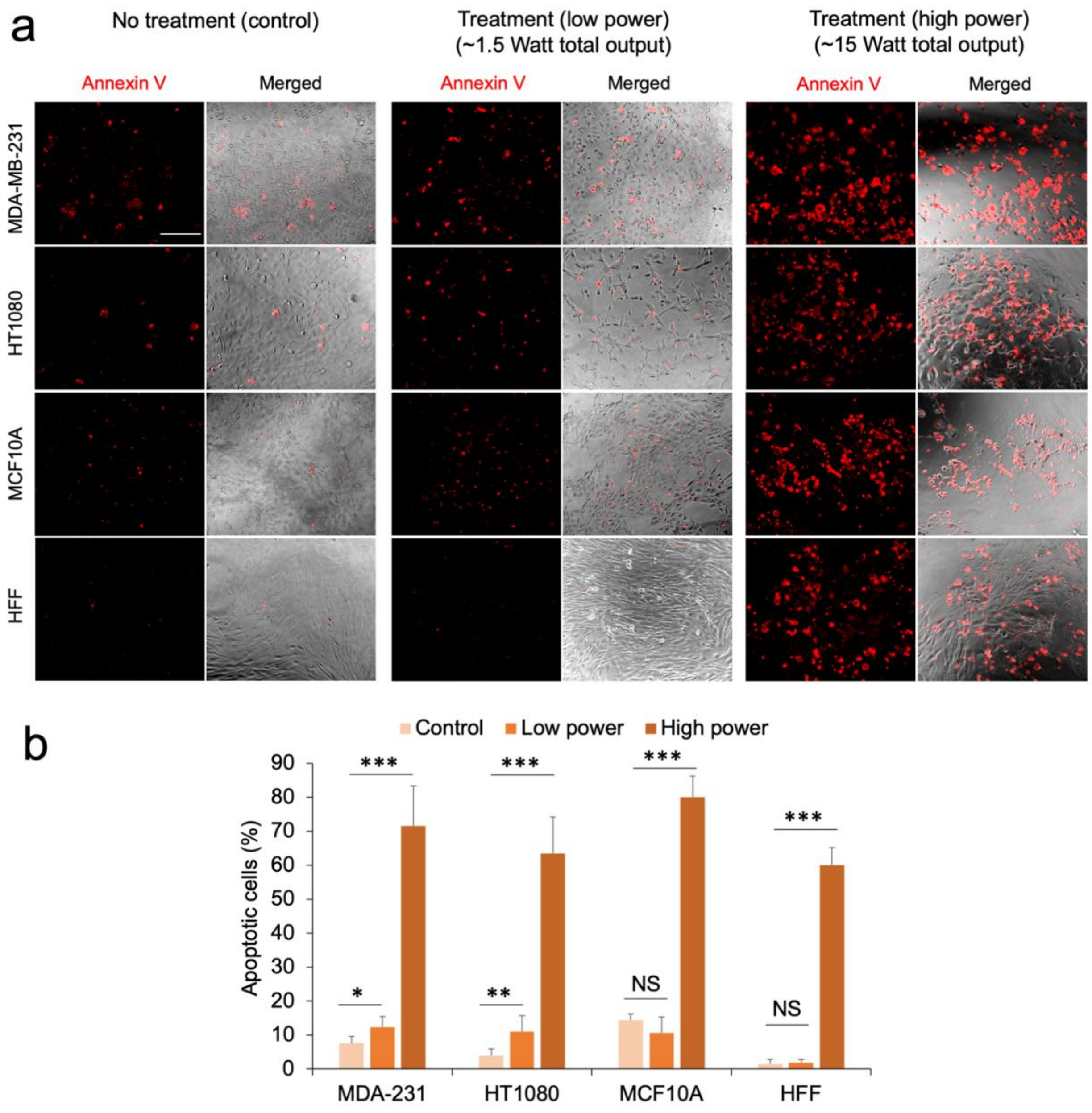

Figure S4. Ultrasound-mediated apoptosis depends on power levels. a) Representative images showing the level of apoptosis in tumor cells (MDA-MB-231, A375p) and normal cells (MCF10A, HFF) at different power levels after 2h treatment. Scale bar: 100 µm. b) Bar diagram illustrating apoptosis level in tumor and normal cells at different power levels. n>1000 cells. Data represent two independent experiments, ANOVA test, $p^{*} < 0.05$, $p^{**} < 0.01$ and $p^{***} < 0.001$.

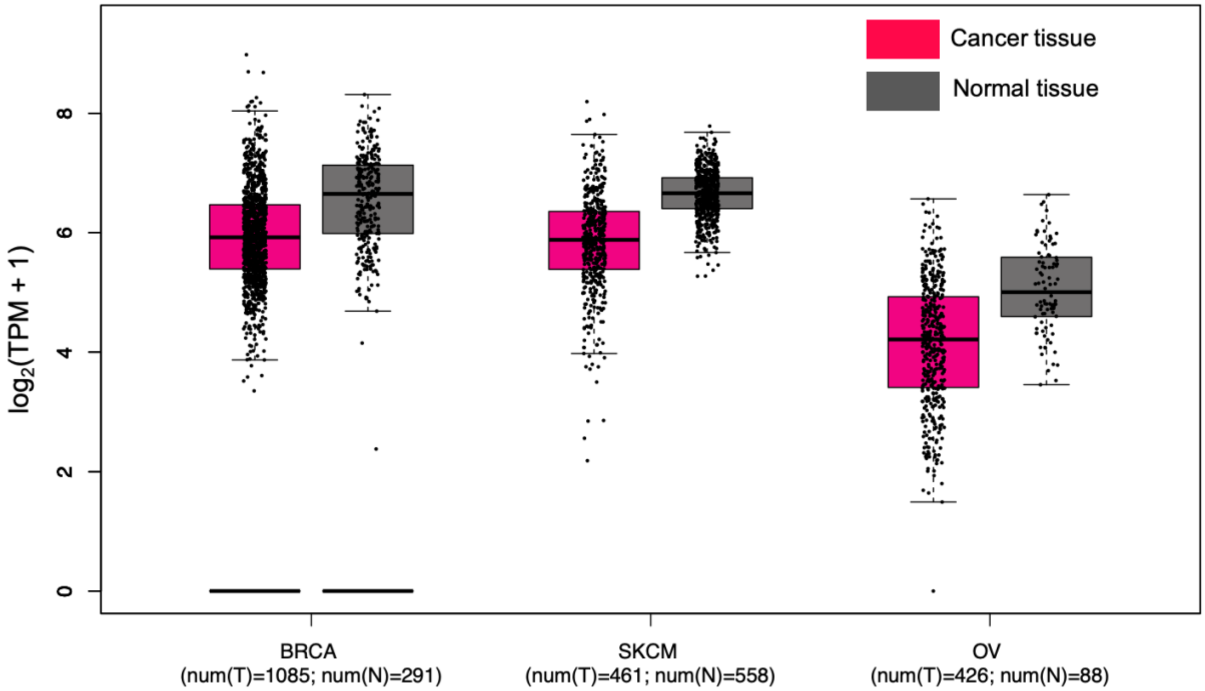

Figure S5. TCGA+GTEx data illustrating the expression level of the Piezo1 gene in breast carcinoma (BRCA), skin melanoma (SKCM) and ovarian adenocarcinoma (OV) and their respective normal tissues. No significant difference in *p* values. They were analyzed by the GEPIA2 tool.

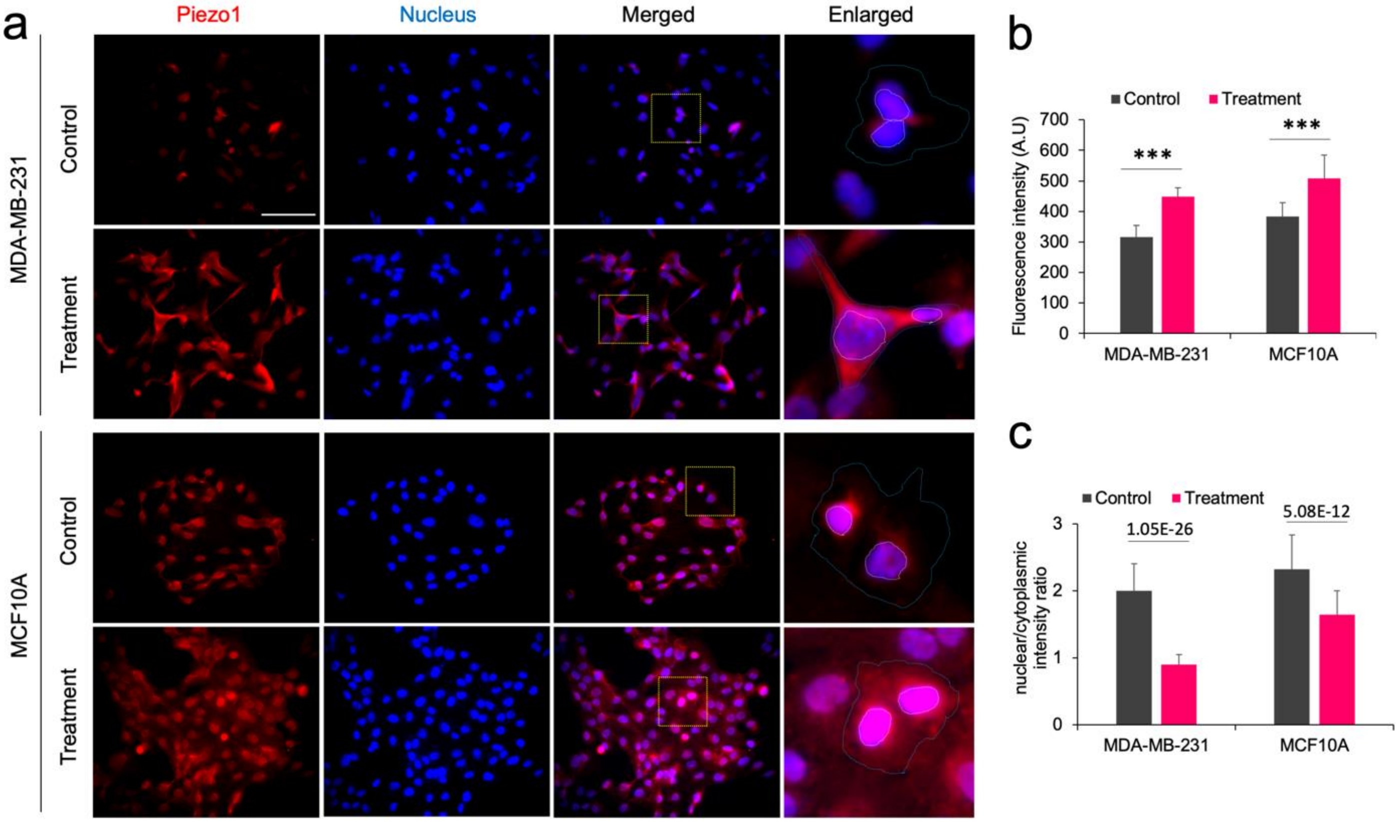

Figure S6. Ultrasound promotes Piezo1 localization to the plasma membrane in tumor cells. a) Representative images displaying Piezo1 expression in MDA-MB-231 and MCF10A cells with and without treatment. Scale bar: 100 µm. b) Bar diagram showing Piezo1 fluorescence intensity profile in tumor and normal cells with and without treatment, n>10 image fields, ANOVA test, p***<0.001. c) Bar diagram illustrating the nuclear/cytoplasmic intensity ratio of Piezo1 in tumor and normal cells with and without treatment, n>55 cells, ANOVA test, p***<0.001.

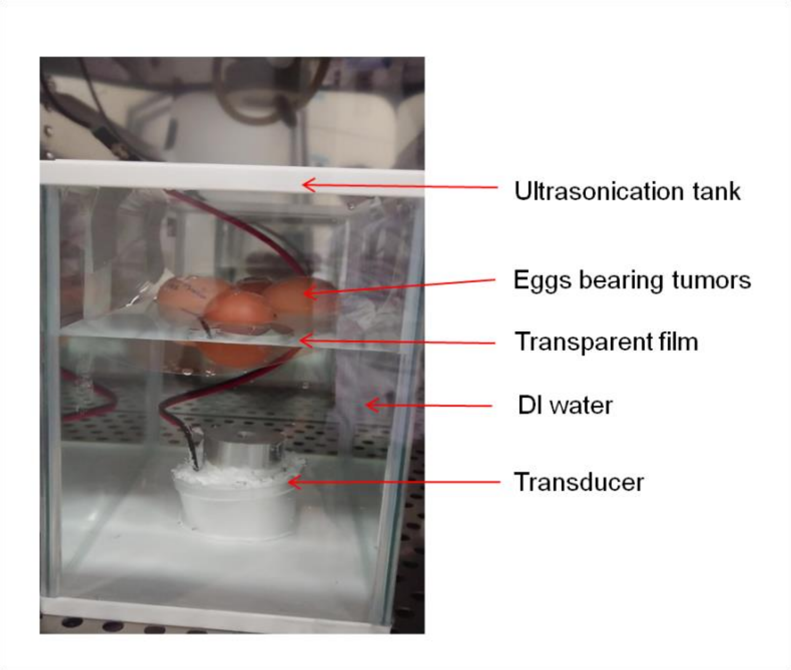

Figure S7. Picture displaying ultrasound tank containing fertilized eggs. Fertilized eggs bearing tumors were half submerged in water using a transparent film and mounted above the ultrasound transducer for treatment.

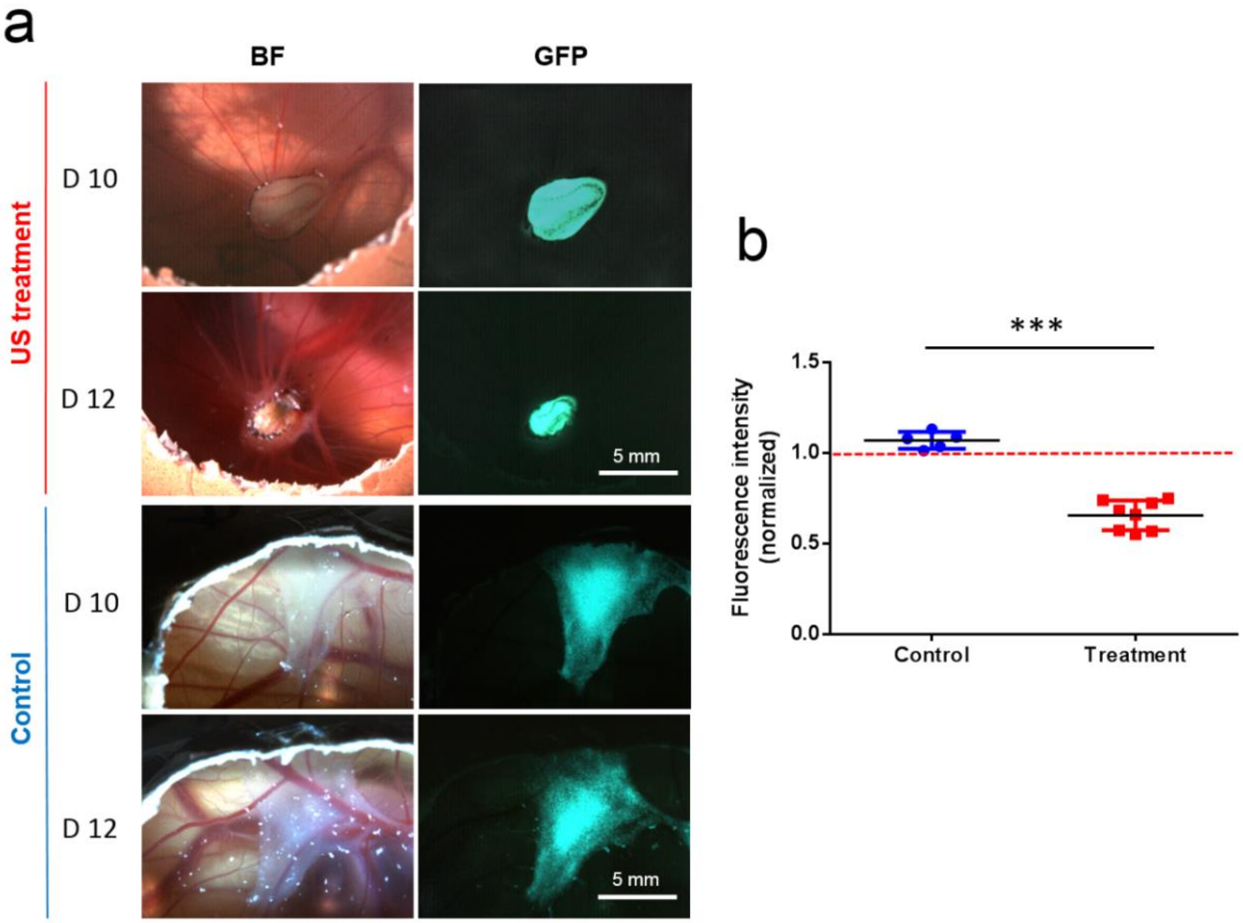

Figure S8. Ultrasound (33 kHz) causes a reduction in HEK293T tumor growth. a) Top two panel rows display bright-field and GFP images of the tumor before (D10) and after two rounds (D12) of treatment. The bottom panel rows show bright-field and GFP images of the tumor without ultrasound treatment on D10 and D12. b) Graph showing fluorescence intensity of tumors with and without US treatment on D12. A red dotted line shows baseline intensity. (n=5 for control and n= 8 for treatment) data represent two independent experiments, p***< 0.001, student t-test.

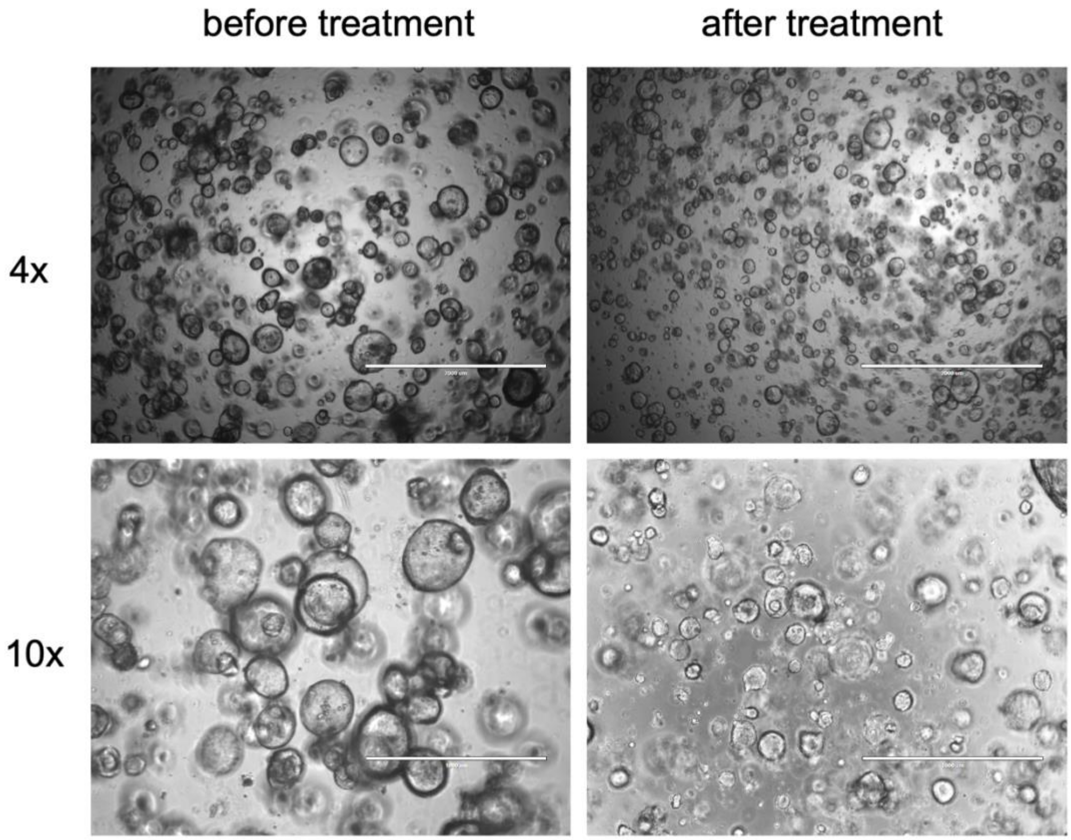

Figure S9. Ultrasound treatment (33 kHz) disrupts pancreatic tumor organoids. Representative images showing organoid morphology before and after ultrasound treatment at 4x and 10x magnification. Scale bar: 2 mm (4x) and 1 mm (10x)

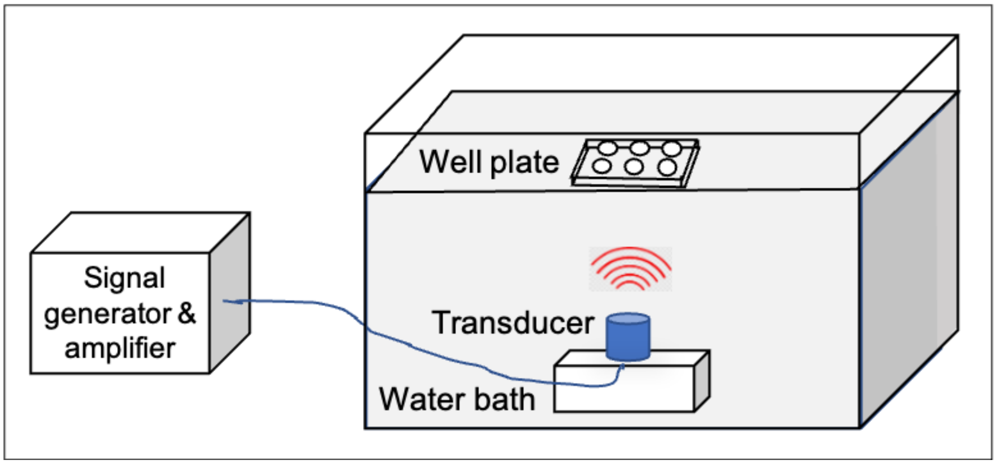

Figure S10. Schematic showing the ultrasound system used for cell/organoid/CAM model treatment.

US Parameters	*In vitro* Models	Animal studies
Frequency	33 kHz	39 kHz
Power	39 mW/cm^2, (200V)	39 mW/cm^2, (200V)<br>51.5 mW/cm^2, (300V)<br>166 mW/cm^2, (400V)
Duty cycle	50%	50%
Treatment duration	2 h	1 h

Table S1. Table elaborating the optimized US parameter used during *in vitro* and animal studies.
